# Complete genome sequence of *Oscillibacter valericigenes* Sjm18-20^T^ (=NBRC 101213^T^)

**DOI:** 10.4056/sigs.2826118

**Published:** 2012-07-30

**Authors:** Yoko Katano, Shun Fujinami, Akatsuki Kawakoshi, Hidekazu Nakazawa, Syoko Oji, Takao Iino, Akio Oguchi, Akiho Ankai, Shigehiro Fukui, Yasuyuki Terui, Sachi Kamata, Takeshi Harada, Satoshi Tanikawa, Ken-ichiro Suzuki, Nobuyuki Fujita

**Affiliations:** 1 Biological Resource Center, National Institute of Technology and Evaluation, Shibuya, Tokyo, Japan; 2 Biological Resource Center, National Institute of Technology and Evaluation, Kisarazu, Chiba, Japan; 3 Tohoku Regional Office, National Institute of Technology and Evaluation, Sendai, Miyagi, Japan

**Keywords:** strict anaerobe, mesophile, valerate-producing, oscillatory motility, alimentary canal, Japanese corbicula clam

## Abstract

*Oscillibacter valericigenes* is a mesophilic, strictly anaerobic bacterium belonging to the clostridial cluster IV. Strain Sjm18-20^T^ (=NBRC 101213^T^ =DSM 18026^T^) is the type strain of the species and represents the genus *Oscillibacter* Iino *et al.* 2007. It was isolated from the alimentary canal of a Japanese corbicula clam (*Corbicula japonica*) collected on a seacoast in Shimane Prefecture in Japan. Phylogenetically, strain Sjm18-20^T^ is closest to uncultured bacteria in digestive tracts, including the enriched cells thought to represent *Oscillospira guilliermondii* Chatton and Perard 1913. The isolated phylogenetic position and some distinct characteristics prompted us to determine the complete genome sequence. The 4,410,036 bp chromosome and the 60,586 bp plasmid were predicted to encode a total of 4,723 protein-coding genes.

## Introduction

**Figure 1:**
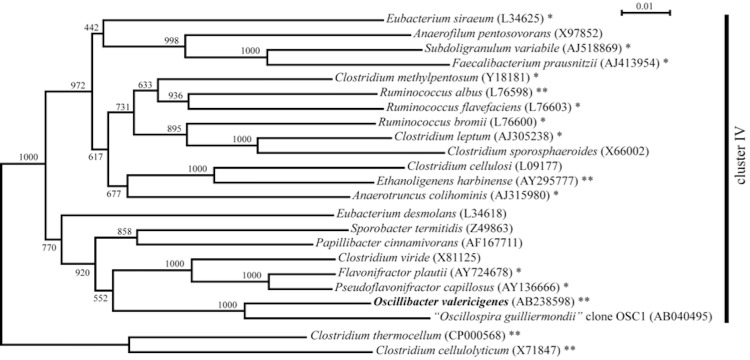
Phylogenetic tree highlighting the position of *O. valericigenes* strain Sjm18-20^T^ relative to other representative type strains within the clostridial cluster IV. The tree was constructed by the neighbor-joining method [4] based on an alignment of 1,339 bp 16S rRNA gene sequences. Corresponding INSDC accession numbers are shown in parentheses. Numbers at nodes indicate support values obtained from 1,000 bootstrap replications. Type strains within the clostridial cluster III were used as the outgroup. Species for which draft assembly sequences are available are labeled with one asterisk, while species for which complete genome sequences are available are labeled with two asterisks. INSDC accession numbers for draft and complete genome sequences are as follows: *A. colihominis*, ABGD00000000; *C. leptum*, ABCB00000000; *C. methylpentosum*, ACEC00000000; *E. harbinense*, CP002400; *E. siraeum*, ABCA00000000; *F. prausnitzii*, ACOP00000000; *F. plautii*, AGCK00000000; *O. valericigenes*, AP012044; *P.capillosus*, AAXG00000000; *R. albus*, CP002403; *R. bromii*, FP929051; *R. flavefaciens*, ACOK00000000; *S. variabile*, ACBY00000000; *C. cellulolyticum*, CP001348; *C. thermocellum*, CP000568.

Strain Sjm18-20^T^ (=NBRC 101213^T^ =DSM 18026^T^) is the type strain of the species *Oscillibacter valericigenes*, which is the type species of the monotypic genus *Oscillibacter* Iino *et al.* 2007 [1]. The strain was isolated from the alimentary canal of a Japanese corbicula clam (*Corbicula japonica*) collected on a seacoast in Shimane Prefecture in Japan. The strain belongs to clostridial cluster IV, one of the 19 clusters of clostridial bacteria proposed based on 16S rRNA gene sequences [2]. Clostridial cluster IV includes phenotypically heterogeneous bacteria, most of which were isolated from digestive tracts and feces of various organisms as well as from anaerobic sewage sludge. While draft genome sequences of some species affiliated with cluster IV, including those obtained as reference sequences for human microbiome projects, have been published, complete genome sequences are thus far been limited to those of a ruminal cellulolytic bacterium *Ruminococcus albus* 7 [3] and an ethanologenic sludge bacterium *Ethanoligenens harbinense* YUAN-3, both of which are phylogenetically distant from *O. valericigenes* with 16S sequence similarities of 86% and 84%, respectively. In addition, the 16S rRNA gene sequence of strain Sjm18-20^T^ is distantly related to the other species within the cluster (Figure 1), with similarity values less than 91%. Instead, the 16S gene sequence is most similar to those of uncultured bacteria in digestive tracts and feces of both herbivorous and omnivorous animals. The latter include the large cells enriched from sheep rumen contents by flow cytometric sorting, which are thought to represent *Oscillospira guilliermondii* Chatton and Perard 1913 [5]. Although *O. guilliermondii* was first described nearly a century ago [6] and has attracted much attention due to its conspicuous morphology, growth in pure culture has not been achieved. In the current NCBI taxonomy database [7], the family *Oscillospiraceae* Peshkoff 1940 [8] is tentatively classified within the order *Clostridiales* to accommodate *O. valericigenes* and related environmental samples, although there is no type strain for the type species *Oscillospira guilliermondii*. Accordingly, the strain Sjm18-20^T^ is currently the only strain in this family having a validly published name.

## Organism information

Strain Sjm18-20^T^ is a mesophilic, neutrophilic, strictly anaerobic bacterium with features as summarized in Table 1 [1]. Unlike other clostridial bacteria, which are typically characterized as being low G+C content, Gram-positive, endospore-forming and anaerobic, Sjm18-20^T^ is Gram-stain negative and non-sporulating. Cells are straight to slightly curved rods with 0.4-0.6 × 2.5-6.0 μm in size. Cells are elongated after prolonged cultivation and often reach 30 μm in length. Optimum growth is observed at 30°C and pH 6.0-6.5. The strain tolerates up to 4% NaCl, but growth is also observed in the absence of NaCl.

**Table 1:** Classification and general features of *Oscillibacter valericigenes* Sjm18-20^T^

**MIGS ID**	**Property**	**Term**	**Evidence code**^a^
		Domain *Bacteria*	TAS [9]
		Phylum *Firmicutes*	TAS [10-12]
		Class *Clostridia*	TAS [13,14]
	Current classification	Order *Clostridiales*	TAS [15,16]
		Family *Oscillospiraceae*	NAS [15,17]
		Genus *Oscillibacter*	TAS [18]
		Species *Oscillibacter valericigenes*	TAS [1]
		Type strain Sjm18-20	TAS [1]
	Gram stain	negative	TAS [1]
	Cell shape	straight or slightly curved rods	TAS [1]
	Motility	oscillatory motility by peritrichous flagella	TAS [1]
	Sporulation	non-sporulating	TAS [1]
	Temperature range	15-35°C	TAS [1]
	Optimum temperature	30°C	TAS [1]
	Carbon source	yeast extract, polypeptone	TAS [1]
	Energy source	heterotrophic	TAS [1]
	Terminal electron receptor		
MIGS-6	Habitat	alimentary canal of Japanese corbicula clam (*Corbicula japonica*)	TAS [1]
MIGS-6.3	Salinity	0-4% NaCl (no growth in 6%)	TAS [1]
MIGS-22	Oxygen	strictly anaerobic	TAS [1]
MIGS-15	Biotic relationship	free-living	NAS
MIGS-14	Pathogenicity	none	NAS
MIGS-4	Geographic location	sea coast in Shimane Pref., Japan	TAS [1]
MIGS-5	Sample collection time	November 10, 2004	NAS
MIGS-4.1	Latitude		
MIGS-4.2	Longitude	NAS	
MIGS-4.3	Depth	NAS	
MIGS-4.4	Altitude	NAS	

a) Evidence codes - TAS: Traceable Author Statement (i.e., a direct report exists in the literature); NAS: Non-traceable Author Statement (i.e., not directly observed for the living, isolated sample, but based on a generally accepted property for the species, or anecdotal evidence). These evidence codes are from the Gene Ontology project [19].

Cells are motile with oscillatory movements. Electron microscopic observation demonstrated the presence of peritrichous flagella [1]. In agreement with this observation, the genome encodes genes necessary for flagellar synthesis and chemotaxis, as is typical in many Gram-positive bacteria. In contrast, while some clostridial bacteria, including the pathogenic species *Clostridium perfringens,* are known to utilize type IV pili for their gliding motility [20], neither genes encoding the constituents of type IV pili, nor the *gld* motility genes of *Flavobacterium johnsoniae* [21], were found in the Sjm18-20^T^ genome, suggesting that flagella are solely responsible for the oscillatory movements.

Strain Sjm18-20^T^ grows poorly even in the medium supplemented with 0.5% each of yeast extract and polypeptone, with a generation time of 18.3 hours under optimum growth conditions [1]. From the genome sequence, strain Sjm18-20^T^ seems to be able to synthesize most amino acids, with the exception of branched-chain amino acids. The genome encodes, however, several ABC transporters possibly involved in the uptake of branched-chain amino acids (OBV_11160-11200, OBV_36860-6900 and OBV_40040-40050).

The strain grows fermentatively and produces acids from D-glucose, L-arabinose, D-ribose and D-xylose, with *n*-valeric acid being the major end product from glucose [1]. Consistent with these observations, genes encoding catabolic enzymes and possible transporters for these sugars could be assigned on the genome. However, we could not identify a gene encoding the authentic form of enolase (EC 4.2.1.11), which catalyzes the penultimate step of glycolysis. Trials of genomic PCR using degenerate primers designed based on the enolase gene sequences of related clostridial bacteria also failed (data not shown). Considering the fermentative phenotype of the strain, either an alternative enolase-like enzyme or a novel metabolic pathway which directs the glycolytic flow towards the synthesis of pyruvate could be present.

Phylogenetic analysis based on 16S rRNA gene sequences unequivocally placed strain Sjm18-20^T^ within the clostridial cluster IV [1] (Figure 1). In addition, phylogenetic analysis based on protein-coding genes such as *ileS*, *valS*, *gyrB* and *rplKLM*, which were extracted from genomic sequences, consistently placed strain Sjm18-20^T^ within the cluster IV (data not shown).

## Genome sequencing information

### Genome project history

*O. valericigenes* Sjm18-20^T^ was selected for sequencing because of its isolated phylogenetic position and characteristics which distinguish this strain from other described clostridial species. Table 2 presents the project information and its association with MIGS version 2.0 compliance [25].

**Table 2:** Project information

**MIGS ID**	**Property**	**Term**
MIGS-31	Finishing quality	Finished
MIGS-28	Libraries used	Three genomic libraries; two plasmid libraries with average insert sizes of 1.7kb and 4.6kb and a fosmid library with average insert size of 40 kb
MIGS-29	Sequencing platforms	ABI 3730xl
MIGS-31.2	Fold coverage	10.2 ×
MIGS-30	Assemblers	Phrap [22,23]
MIGS-32	Gene calling method	Glimmer3 [24]
	INSDC ID	AP012044.1 (chromosome) AP012045.1 (plasmid pOBV01)
	Genbank Date of Release	Oct 1, 2011
	NCBI project ID	41823
	GOLD ID	Gc01995
MIGS-13	Source material identifier	NBRC 101213
	Project relevance	biotechnology, systematics

### Growth conditions and DNA isolation

*O. valericigenes* Sjm18-20^T^ cells were grown in a 200 ml volume at 30°C under N_2_ atmosphere in GYP medium in which air had been replaced with nitrogen gas by flushing [1]. DNA was isolated from 1 g of wet cells by manual extraction after lysis with lysozyme and SDS.

### Genome sequencing and assembly

The genome of *O. valericigenes* Sjm18-20^T^ was sequenced using the conventional whole-genome shotgun sequencing method. DNA shotgun libraries with average insert sizes of 1.7kb and 4.6kb were generated in pUC18 (TaKaRa), while a fosmid library with average insert size of 40 kb was constructed in pCC1FOS (EPICENTRE) as described previously [26]. A total of 37,824 clones (20,352, 12,288 and 5,184 clones from libraries with 1.7kb, 4.6 kb and 40 kb inserts, respectively) were subjected to sequencing from both ends of the inserts on ABI 3730xl DNA Analyzer (Applied Biosystems). Sequence reads were trimmed at a threshold of 20 in Phred score and assembled by using Phrap and CONSED assembly tools [22,23]. For alignment and validation of contigs, Optical Mapping (OpGen) was used. Gaps between contigs were closed by sequencing PCR products which bridge two neighboring contigs. Finally, each base of the genome was ensured to be sequenced from multiple clones either from both directions with Phrap quality score≧70 or from one direction with Phrap quality score ≧40.

### Genome annotation

Complete sequences of the chromosome and the plasmid were analyzed using Glimmer3 [24] for predicting protein-coding genes, tRNAscan-SE [27] and ARAGORN [28] for tRNA genes, and RNAmmer [29] for rRNA genes. The functions of predicted protein-coding genes were assigned manually, using in-house genome annotation system OCSS (unpublished), from the comparison with Uniprot [30], Interpro [31], HAMAP [32] and KEGG [33] databases.

## Genome properties

**Figure 2:**
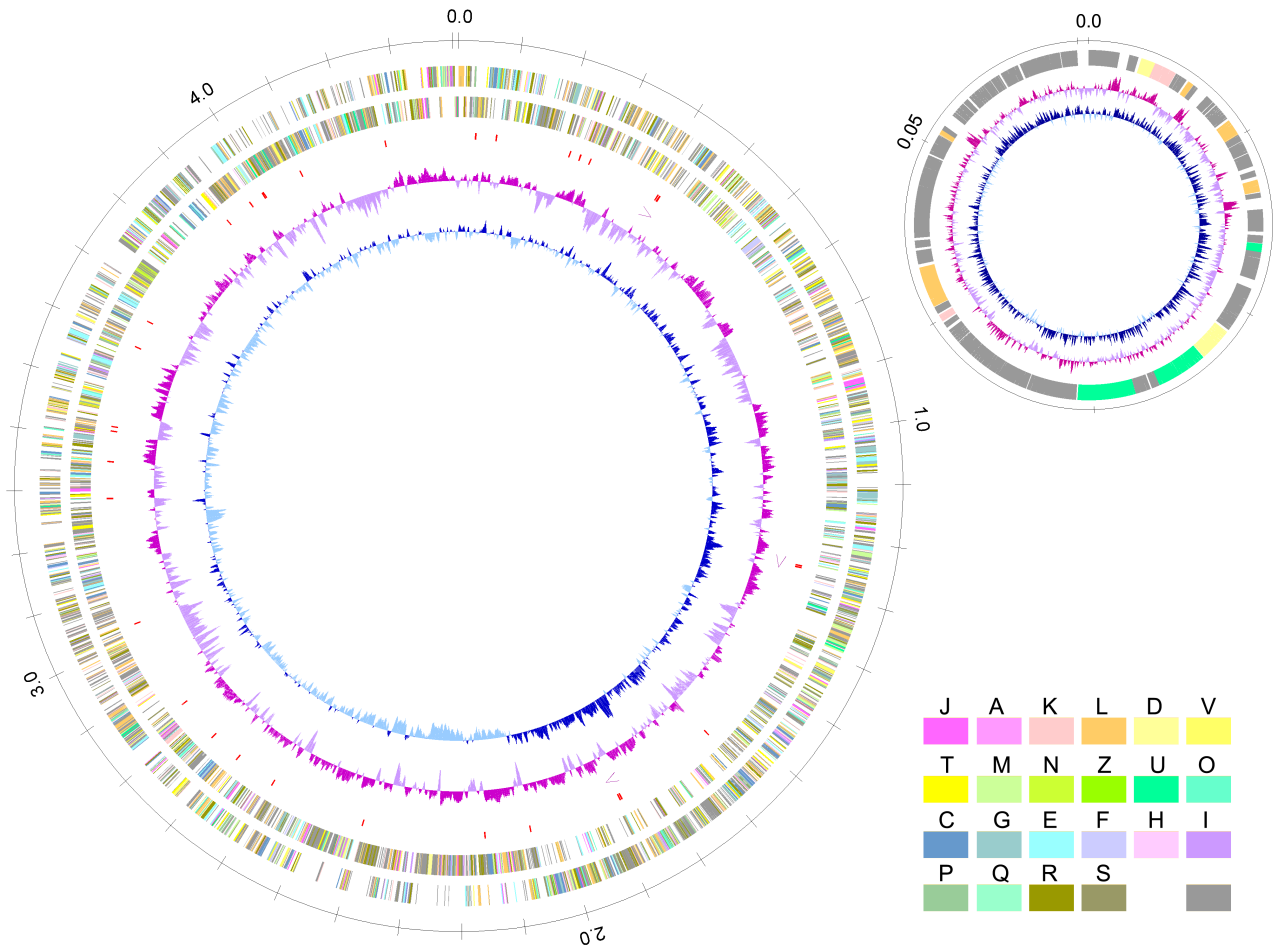
Circular representation of the *O. valericigenes* Sjm18-20^T^ chromosome and the plasmid. From outside to the center: circles 1 and 2, predicted protein coding genes on the forward and reverse strands, respectively; circle 3, tRNA genes; circle 4, rRNA operons; circle 5, G+C content; circle 6, GC skew. Predicted protein coding genes are colored according to their assigned COG functional categories (see Table 4).

The genome of *O. valericigenes* Sjm18-20^T^ consisted of a circular chromosome of 4,410,036 bp and a circular plasmid of 60,586 bp (Figure 2). The chromosome was predicted to contain 4,656 protein-coding genes, 58 tRNA genes, 9 rRNA genes and 5 other RNA genes, whereas the plasmid contained 67 predicted protein-coding genes. Of the total of 4,723 protein-coding genes predicted in the genome, 2,483 (52.6%) were assigned known functions, 1,499 (31.7%) were similar to genes with unknown function in other bacterial genomes, and 741 (15.7%) had no similarity with other genes. Average G+C contents of the chromosome and the plasmid were 53.3% and 43.3%, respectively. The properties and the statistics of the genome are summarized in Tables 3-4.

**Table 3:** Nucleotide content and gene count levels of the genome

Attribute	Value	% of total^a^
Genome Size (bp)	4,470,622	100.0%
Coding region (bp)	3,837,687	85.8%
G+C content (bp)	2,377,733	53.2%
Number of replicons	1	
Extrachromosomal elements	1	
Total genes	4,795	
RNA genes	72	
rRNA operons	3	
Protein-coding genes	4,723	100.0%
Pseudo genes	0	0.0%
Genes with function prediction	2,483	52.6%
Genes in paralog clusters	1,666	35.3%
Genes assigned to COGs	3,037	64.3%
Genes assigned Pfam domains	3,007	63.7%
Genes with signal peptides	1,064	22.5%
Genes with transmembrane helices	1,002	21.2%
Paralogous groups	500	% of totala

a) The total is based on either the size of the genome in base pairs or the total number of protein coding genes in the annotated genome.

**Table 4:** Number of genes associated with the 25 general COG functional categories

**Code**	**Value**	**% age**^a^	**Description**
J	160	3.39	Translation
A	1	0.02	RNA processing and modification
K	375	7.94	Transcription
L	419	8.87	Replication, recombination and repair
B	0	0.00	Chromatin structure and dynamics
D	47	1.00	Cell cycle control, mitosis and meiosis
Y	0	0.00	Nuclear structure
V	92	1.95	Defense mechanisms
T	222	4.70	Signal transduction mechanisms
M	116	2.46	Cell wall/membrane biogenesis
N	61	1.29	Cell motility
Z	1	0.02	Cytoskeleton
W	0	0.00	Extracellular structures
U	60	1.27	Intracellular trafficking and secretion
O	81	1.72	Posttranslational modification, protein turnover, chaperones
C	224	4.74	Energy production and conversion
G	150	3.18	Carbohydrate transport and metabolism
E	284	6.01	Amino acid transport and metabolism
F	77	1.63	Nucleotide transport and metabolism
H	103	2.18	Coenzyme transport and metabolism
I	79	1.67	Lipid transport and metabolism
P	125	2.65	Inorganic ion transport and metabolism
Q	34	0.72	Secondary metabolites biosynthesis, transport and catabolism
R	370	7.83	General function prediction only
S	240	5.08	Function unknown
-	1,686	35.70	Not in COGs

The total is based on the total number of protein coding genes in the annotated genome.

## Similarities to *O. guilliermondii*

Phylogenetic analysis based on 16S rRNA gene sequences revealed that *O. valericigenes* Sjm18-20^T^ is closely related to the uncultivated cells thought to represent *Oscillospira guilliermondii* Chatton and Perard 1913 [5]. In addition, strain Sjm18-20^T^ shares some phenotypic characteristics, including the elongated and often curved cell morphology, the oscillatory motility by means of peritrichous flagella, and the Gram-negative staining, with those described for *O. guilliermondii* [1]. However, while *O. guilliermondii* was reported to form endospores, spore formation was not detected with *O. valericigenes* Sjm18-20^T^ by microscopic observations and by heat treatment for testing the presence of heat resistant bodies such as spores. In phylogenetically related bacteria such as *Bacillus* and *Clostridium*, phosphorylation of Spo0A, a master regulatory factor, is known to initiate the process of sporulation through the successive synthesis of sporulation-stage specific sigma factors. We found that the genome of strain Sjm18-20^T^ encoded the Spo0A factor (OBV_15500) and all sporulation sigma factors known in other bacteria, i.e., sigma H (OBV_22080), sigma E (OBV_21490), sigma F (OBV_29180), sigma K (OBV_12200), and sigma G (OBV_24420), as well as other regulatory proteins related to the sigma cascade. In contrast, genes necessary for the later stages of sporulation, i.e., the formation of cortex and spore coat, seemed either largely different or partly missing. For example, *cotF*, *cotS* and *yabQ* genes, widely found in the genomes of clostridial species, could not be found in the genome of strain Sjm18-20^T^. Strain Sjm18-20^T^ might have the potential for sporulation, although it needs to be further investigated whether conditions exist under which this bacterium would actually sporulate.
